# The management of acute myocardial infarction in the Russian Federation: protocol for a study of patient pathways

**DOI:** 10.12688/wellcomeopenres.12478.2

**Published:** 2018-04-06

**Authors:** Anna Kontsevaya, Natalia Bobrova, Olga Barbarash, Dmitry Duplyakov, Alexey Efanov, Albert Galyavich, Maria Frants, Larisa Khaisheva, Tatyana Malorodova, Olga Mirolyubova, Andrei Nedbaikin, Irina Osipova, Dmitry Platonov, OIga Posnenkova, Liudmila Syromiatnikova, Katie Bates, David A Leon, Martin McKee

**Affiliations:** 1 Department of Non-Communicable Disease Epidemiology, National Research Center for Preventive Medicine, Moscow, Russian Federation; 2 London School of Hygiene and Tropical Medicine, London, UK; 3 Federal State Budgetary Scientific Institution Research Institute for Complex Issues of Cardiovascular Diseases, Kemerovo, Russian Federation; 4 Samara Regional Cardiology Dispensary, Samara, Russian Federation; 5 Tyumen Scientific Practical Center, Tyumen, Russian Federation; 6 Cardiology Department, Kazan State Medical University, Kazan, Russian Federation; 7 Department of Outpatient Cardiology, District Clinical Hospital, Khanty-Mansiysk, Russian Federation; 8 Department of Therapy of The Faculty Of Advanced Training And Professional Retraining, Rostov State Medical University, Rostov-on-Don, Russian Federation; 9 Department of Pharmacology, Belgorod State National Research University, Belgorod, Russian Federation; 10 Department of Internal Medicine, Northern State Medical University, Arkhangelsk, Russian Federation; 11 Bryansk Regional Cardiology Hospital, Bryansk, Russian Federation; 12 Faculty Therapy Department, State Educational Institution, Altay State Medical University, Barnaul, Russian Federation; 13 Department of Internal Medicine, Tver State Medical University, Tver, Russian Federation; 14 Department of Novel Cardiology Information Technologies, Saratov State Medical University n.a. V.I. Razumovsky, Saratov, Russian Federation; 15 Regional Vascular Center of State Autonomous Institution of Perm Region, Clinic Hospital №4, Perm, Russian Federation; 16 The Arctic University of Norway, Tromsø, Norway

**Keywords:** myocardial infarction, health seeking behaviour, health services research, patient pathways

## Abstract

**Background**: Death rates from cardiovascular disease in Russia are among the highest in the world. In recent years, the Russian government has invested substantially in the healthcare system, with a particular focus on improving access to advanced technology, especially for acute myocardial infarction (AMI). This protocol describes a study to understand the management of AMI in different Russian regions, investigating the role of patient, clinical, and health system characteristics.

**Methods:** A prospective observational study has recruited a representative sample of AMI patients within 16 hospitals from 13 regions across Russia. Criteria for inclusion are being aged 35-70 years with a confirmed diagnosis of AMI and surviving until the day after admission. Information being collected includes health system contacts and features of clinical management prior to the event and in the 12 months following discharge from hospital. Following initial exploration of the data to generate hypotheses, multivariate analyses will be applied to assess the role of these characteristics in both treatment decisions and any delays in time critical interventions. Between June 2015 and August 2016, 1,122 patients have been recruited at baseline and follow-up to 12 months post-discharge is scheduled to be completed by autumn 2017. The study is unique in examining patient factors, clinical management prior to admission and in hospital in the acute phase and throughout the critical first year of recovery across a diverse range of geographies and facilities. It uses standardized instruments to collect data from patients and health care providers and includes regions that are diverse in terms of geography and development of cardiology capacity. However, given the limited health services research capacity in the Russian Federation, it was not possible to obtain a sample that was truly nationally representative.

## Introduction

The Russian Federation has one of the highest burdens of cardiovascular disease (CVD) in the world ^1^, an important cause of its low life expectancy at birth, only 70.5 years in 2015 according to the World Health Organization. There are many potential reasons for the very high CVD rates, spanning the entire causal pathway, from underlying social determinants of health ^2^ through proximal risk factors such as smoking, diet, hazardous alcohol consumption ^3^, to weaknesses in the health care system ^4^. Consequently, a comprehensive response requires actions at all levels. In this study, we focus our attention on the contribution of the health system.

Although the Soviet Union initially placed a high priority on health, a combination of economic weakness ^5^, isolation from international developments ^6^, and the considerable challenges of delivering modern healthcare to a vast country, meant that the system inherited by the newly independent Russian Federation lagged behind that in many western countries. The scale of the challenge was illustrated by a study that compared levels of mortality amenable to health care, which showed that the sustained improvements seen in the west after the mid-1960s were not achieved in the Soviet Union ^4^.

Although the inherited system was extensive and well-staffed in comparison to other middle-income countries, it struggled initially to adopt the rapid advances that were taking place in medical science, especially in areas such as the management of acute myocardial infarction(AMI), which elsewhere was being transformed by the introduction of thrombolysis and percutaneous transluminal coronary angioplasty (PTCA) ^7, 8^.

These concerns attracted official recognition in 2005 when a national priority project was announced to improve population health ^9^. This had several elements, including better access to high-quality health care, a renewed emphasis on prevention in the health care system, strengthened primary care, and greater provision of advanced medical technology. A further goal to reduce mortality from cardiovascular diseases was added in 2008 ^10^. This was supported by greater funding for salaries of health professionals and new equipment. Forty-five percent of the additional funding was allocated to advanced medical technology ^9^. This has been associated with a marked increase in utilisation of such technology, including PTCA. Yet it is recognised that that outcomes of myocardial infarction still lag behind those in Western countries, although the reasons are not fully understood.

Previous research has used data from a large federal registry, recording details of patients presenting with acute coronary syndrome (ACS) to a network of participating hospitals ^11, 12^. However, while providing some valuable insights, results have been limited as data are collected only on the management of patients in hospital, while it is now recognised that pre- and post-hospital care, including early thrombolysis and secondary prevention also play important roles in reducing mortality ^13^.

There is a clear need to document in detail the management of patients presenting with AMI across Russia. Information is needed on the entire patient experience, from onset of symptoms, through to the phase of acute care to the long-term management and treatment they receive once they have been discharged. This should describe how they are treated, by whom, and whether there are any delays in obtaining treatment, especially at those points in the patient journey that are time-critical events. It should assess whether the treatment provided complies with accepted good practice and assess where problems are found, and identify plausible reasons that can be addressed by changes to policy and practice. Here we present the protocol for a study we are undertaking that does this in hospitals across diverse regions of the Russian Federation. This is part of a large international project seeking to understand the reasons for the high levels and poor outcomes of CVD in Russia.

## Methods and study design

The objective of this paper is to describe the context and design of a study of the management of acute myocardial infarction in Russian hospitals.

### Objectives of the study itself

a) To describe current treatment of AMI in different regions of Russia and in different types of medical facilities accepting patients with AMI, comparing observed practice with that recommended in Russian ^14^ and European guidelines ^15^ so as to identify barriers to effective treatment and continuity of care at different stages of the patient journey - prior to admission, within the hospital, and following discharge to polyclinic cardiologists and general physicians.
b) To describe and, where possible, explain differences in management of patients defined by gender, socio-economic position, and distance from facilities.
c) To propose changes to policy and practice that will remove barriers to effective and timely treatment for all.

The study is, to our knowledge, the first ever study in different parts of Russia describing the pathway followed by patients with AMI. It is thus primarily hypothesis generating, although some testing will be possible. It is observational and seeks to recruit a representative sample of patients presenting with AMI at 16 hospitals in 13 Russian regions and who survive at least until the morning after admission. Data are collected on both the index admission and any encounters with the health system in the preceding 12 months and follow up at 6 and 12 months after discharge.

### Study population and recruitment

The target population is men and women aged 35–75 years, admitted to a hospital or cardiology centre with a presumed diagnosis of AMI that is subsequently confirmed and who survive until the day following admission, including those who subsequently die in hospital and those who are discharged alive. Patients hospitalized in any department or ward with a primary diagnosis of AMI were eligible. The age range was selected for consistency with a major population-based study which we are also conducting looking at aetiological factors and treatment in the Russian cities of Arkhangelsk and Novosibirsk. As noted above, current life expectancy at birth (both sexes combined) in Russia is 70.5 years.

As this study seeks to capture the actual management of patients diagnosed as having an AMI, we have not imposed uniform diagnostic eligibility criteria. Instead we accepted the criteria for AMI used in each centre. These nevertheless all included standard ECG changes and cardiac enzymes (creatinine phosphokinase as minimum). Where they differ was in the use of troponin assays, with the precise version varying. In analyses of the completed dataset, we will explore the extent to which any observed differences in diagnostic criteria impact on which patients are offered treatment, taking as our reference the most inclusive criteria observed in any facility.

The overall inclusion and exclusion criteria are summarized in Box 1.

**Box 1. Inclusion and exclusion criteria**

**Table:** 

**Inclusion criteria** • Acute admissions with a presumed diagnosis of AMI subsequently confirmed by the morning after admission. • Age 35–75, male or female • Living in the oblast/republic in which the hospital is situated (to enable access to previous medical records) • Survived until the day after admission
**Exclusion criteria** • Participating in clinical trials where this is known at the moment of inclusion, as they are likely to be receiving atypical treatment. • Patients, referred from another facility if they have already spent more than 24 hours in it. • AMI occurring in hospital following a surgical procedure

For each facility, recruitment was staggered over the course of 6–9 months. Data are collected by staff and medical students in each centre. However, they have limited time available to do this work, so it was determined that it was feasible for a maximum of 1 patient each day to be recruited. The study timeline is presented in Box 2.

**Box 2. Study timeline**

**Table:** 

Study stage	Period
Recruitment to the study/hospital stage	June 2015–August 2016
6 months follow up	December 2015–February 2917
12 months follow up	July 2016–September 2017
Data analysis and publications	October 2017–February 2019

**Figure 1.**
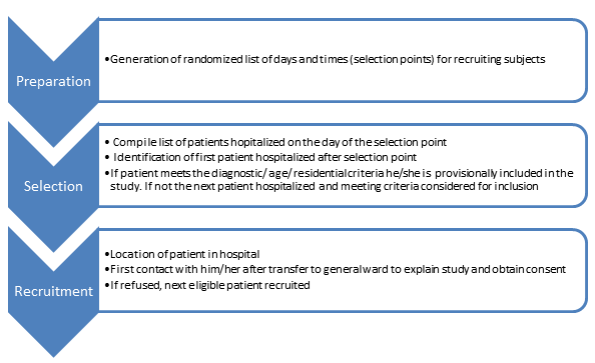
Recruitment procedure used in the study of *management of acute myocardial infarction in Russian Federation*.

In order to recruit as representative sample of AMI cases as possible, the following procedure was used. Within the recruitment period, a list of random dates and times was generated by the central study coordination team, constrained so that, for each facility, none could occur on the same day. On a daily basis, in each facility, the list of all patients admitted during the previous day with a confirmed diagnosis of AMI was compiled. The patient to be recruited was selected from this list as the first to be admitted following the randomly selected date and time. If this patient could not be recruited, the next patient in order of admission was approached until for that day a patient was enrolled in the study. The recruitment process is summarised in Figure 1.

When approached by the study team, a patient was given a verbal explanation of the study, including the importance of follow up, and given an information leaflet. Signed informed consent was sought.

### Selection and characteristics of facilities

**Figure 2.**
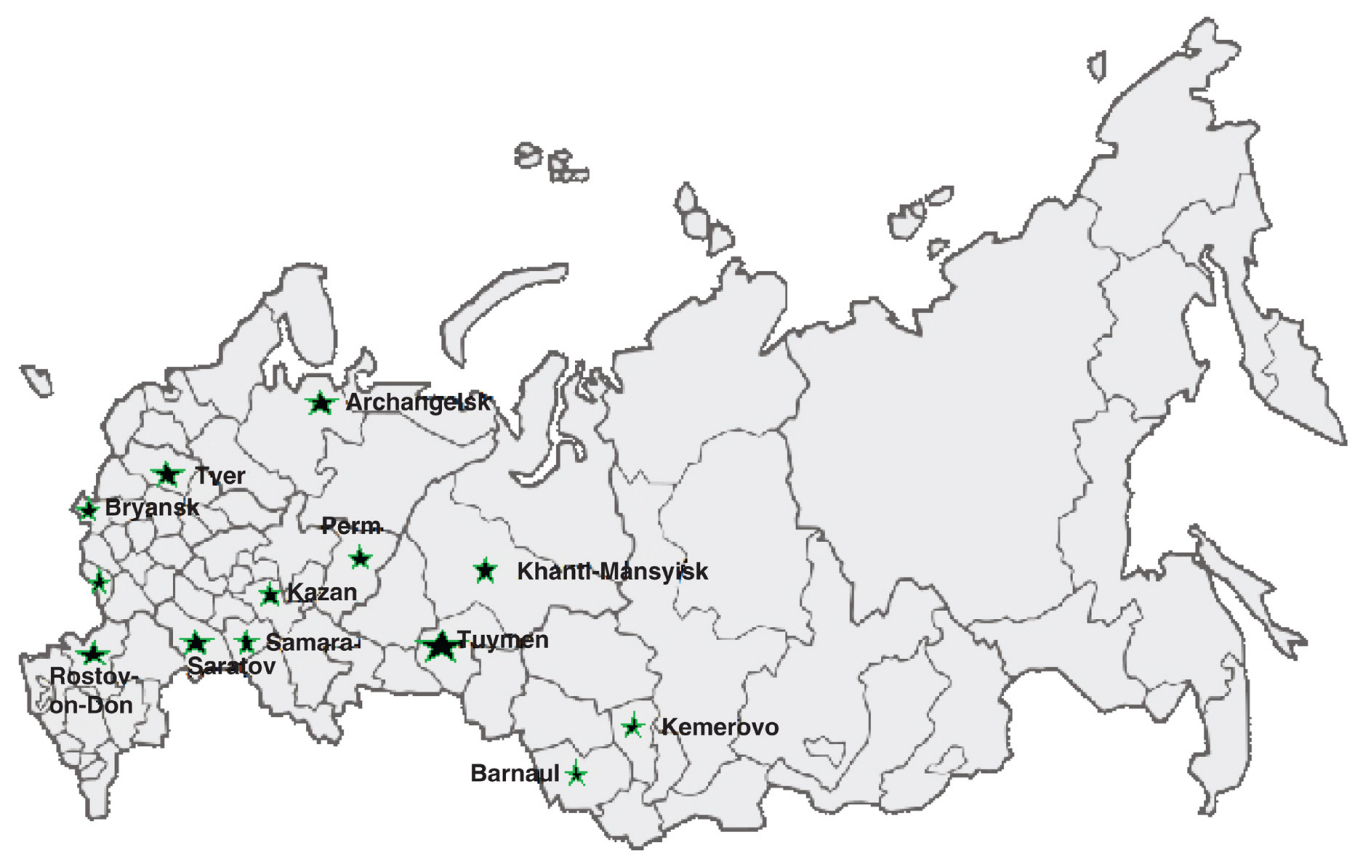
Location of participating centres in the *management of acute myocardial infarction in Russian Federation*.

Patients have been recruited from hospitals in 13 regions across the Russian Federation. Ideally, we would have employed a large, randomly selected sample of facilities but this was not practical as infrastructure for undertaking such research in Russia, including clinicians with relevant research skills, is limited. Consequently, it was necessary to draw a convenience sample, identifying clinicians willing to participate. It is recognised that the settings cannot be entirely representative of the country as the study centres are mainly from European part of the Russian Federation, with few from Siberia. In particular, two are from some of the wealthiest regions, benefitting from large oil and gas reserves, but with a sparse population. However, judged in terms of penetration of advanced treatment, in this case the rate of PTCA per 100,000 population, these regions span almost the entire range seen in the country ( Supplementary File 1, unadjusted crude rates). We explicitly included some small facilities, even though they have limited capacity to intervene. We considered this important to capture as much of the spectrum of treatment, as experienced by patients, as possible. The names and locations of participating centres are shown in Figure 2.

A standardized form was completed describing basic information on capacity and activity in each facility. In most cases, the study included a single facility in each region. The exceptions are Samara region (3 facilities) and Tver (2 facilities). Five facilities (31.3%) serve cities (municipal hospitals) and 11 (68.8%) are regional facilities. Half are specialized cardiology hospitals and half are general hospitals with cardiology departments.

All facilities can measure troponin or CK-MB measurements at all times of the day and night. Among them, 10 (62.5%) measure troponin I, 5 (31.3%) Troponin T, 5 (31.3%) high sensitivity Troponin I, 2 (12.5%) high sensitivity Troponin T and 13 (81.3%) CK-MB. Echo facilities are available 24 hours and 7 days per week in 9 facilities (56.3%) and in working hours only, Monday-Friday in 7 (43.8%).

Twelve (75%) of the facilities can perform PTCAs, all 24 hours a day; the remaining four are two small municipal hospitals in Samara, the regional cardiology hospital in Bryansk and one of the hospitals in Tver, all of which are able to perform thrombolysis). The mean monthly number of PCTA procedures per interventional cardiologist is 39 (minimum 3, maximum 170). None of the hospitals in this study offer open cardiac surgery.

Nine of the facilities include a rehabilitation department on the same site. All others use separate sanatoria.

Table 1 reports data on activity in the 13 facilities information, and was available derived from returns of the hospitals to the Federal Ministry of Health. This provides contextual data on these hospitals, by calendar year, indicating both the size of the hospital and the total number of patients with an MI. As is apparent, there is considerable variation in levels of activity, patient characteristics and patient outcomes across these 13 hospitals. For example, the proportion of patients with AMI admitted within 24 hours of symptoms ranges from 28.4% to 92.5%.

**Table 1.** Treatment characteristics of clinics from 13 regions for 2015, from official statistical forms – Form 14 “Data on hospital performance”.

	N of beds in clinic. Total;	N of cardiology beds in clinic	N of MI patients in the clinic in 2015	Patients with AMI hospitalized in first 24 hours from symptom onset	In-hospital mortality of patients with AMI	In-hospital mortality of patients with AMI in first 24 hours	Percentage of patients undergoing PTCA (from those, hospitalized in first 24 hours)	Percentage of patents received thrombolysis (from those, hospitalized in first 24 hours)
City hospital N 1, Archangelsk	991	140	558	67.6%	10.9%	4.6%	61.3%	8.2%
Altay regional cardiology hospital, Barnaul	356	356	362	68.4%	8.1%	5.7%	82%	4,5%
Belgorod regional hospital	1055	112	501	75.8%	2.4%	0.4%	87.8%	12.2%
Bryansk cardiology clinic	192	192	254	45.3%	14.6%	7.9%	0	4.3%
Kazan interregional clinic center, Tatarstan	400	92	430	81.4%	4.7%	4.7%	81.4%	1.6%
Kemerovo cardiology clinic	355	218	1032	65.0%	9.5%	4.8%	68.1%	3.9%
Perm city clinic N 4	544	77	1052	83.6%	9.1%	3.5%	66.7%	8.0%
Emergency city hospital, Rostov- on-Don	845	180	1280	28.4%	10.2%	6.1%	20.5%	8.5%
Samara regional cardiology clinic	671	458	2220	92.5%	8.4%	4.8%	43.3%	10.3%
Tver regional hospital	930	160	516	50.0%	4.8%	2.3%	78.5%	25.0%
Tver city hospital	760	345	319	66.1%	9.1%	6.0%	0	24.5%
Tuymen regional hospital	500	150	1100	88,7%	12.4%	4.4%	64.1%	12.0%
Khanty-Mansiysk regional hospital	650	32	184	91.8%	6.0%	0.5%	62.5%	2,7%

### Data collection

**Figure 3.**
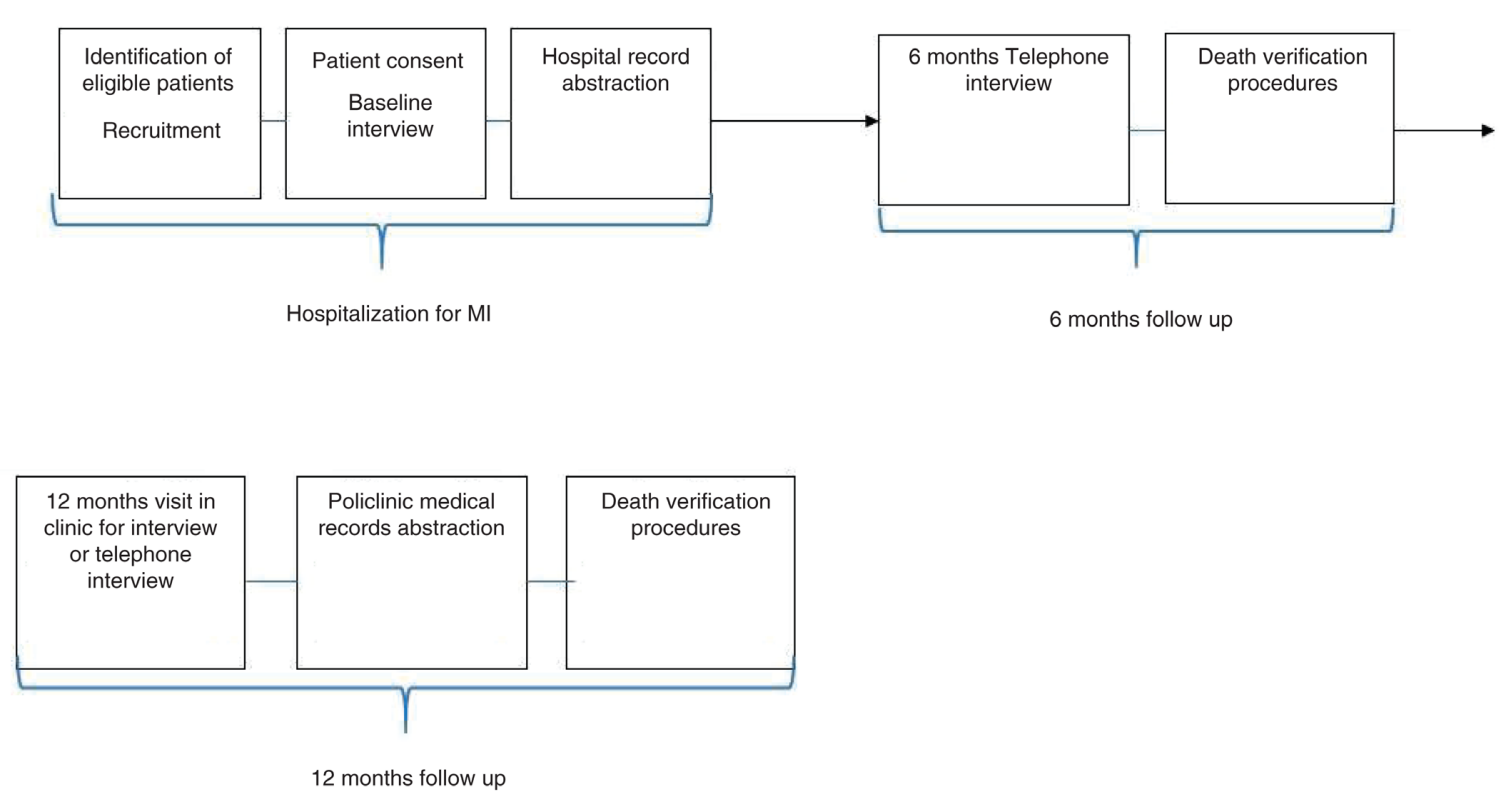
Overview of the study design of *management of acute myocardial infarction in Russian Federation*.

Data are collected from patient interviews at three points: during index admission, at 6 months and at 12 months following discharge. Information is also extracted from medical records with respect to the index admission and contacts with the health system in the 12 month period preceding admission and following discharge, at both the hospital and the polyclinic that the patient attends ( Figure 3). All deaths reported during the 12 months of follow up are verified.

The information collected is designed to shed light on a series of decisions made by both patient (and their family) and health care providers, along the clinical pathway. These decision points arise at every stage of the patient journey, from when the patient develops symptoms, such as whether they recognize the need to seek help, to the decision by the physician responsible for follow up to prescribe secondary prevention medication.

### Management

The co-ordinator at each site, a practicing cardiologist, is responsible for oversight of the project and recruiting a team of interviewers, who are provided with written guidance on the conduct of the study and have received training, co-ordinated from Moscow, via Skype. Most of the data are being entered into a specially designed template created in Microsoft on Access™. However, the 12 month data are being entered using a bespoke data entry interface based on SURVANT [ http://www.survant.net]. The data are checked by the study co-ordinator in Moscow. Data analysis will be conducted using the Stata 14.

### Piloting

The study was piloted in 3 study centres in the spring of 2015, involving all stages of the study. In each centre, a group of 5 patients who were at that point in hospital and two groups of 5 patients each who had been hospitalised in a designated week 6 and 12 months previously were included. For these subjects, questionnaires were completed as was abstracting of medical records. The pilot results were used to refine the study documentation and standard operating procedures. Patients included in the pilots were not included in the main study.

### Patient interviews

Participants are interviewed three times in person: during the index hospitalization and at 6 and 12 months later. A core data set comprises socio-demographic data, an account of events in the period between onset of symptoms and admission, including signs and symptoms, health seeking behaviour, and treatment received ( Box 3). The questionnaires are available as Supplementary File 2 and Supplementary File 3. As far as possible, questions were consistent with those used in previous studies in Russia, such as the Health in Times of Transition (HITT) project ^15^, the Izhevsk Family Study ^16^, and the Russian Longitudinal Monitoring Survey 7 ^17^. Other survey instruments were drafted in English and translated into Russian.

**Box 3. Data items collected at each stage**

**Table:** 

*Components of initial in-hospital interview*
Socio-economic status (education, marital status, employment, economic status) Characteristics and date and time of onset of initial symptoms Patient actions after appearance of initial symptoms, including health seeking behaviour and any delays Patient-reported knowledge of their medical history (e.g. hypertension and cholesterol levels, history of previous cardiac problems), visits to physicians during the previous 12 months and actions taken (BP and cholesterol measurement etc.)) Key behavioural risk factors (smoking and alcohol) Experience of counselling on risk factors Medications prescribed before hospitalization Patient contact details (phone, mobile, email, address)
*Components of follow-up interviews and medical record extraction*
Employment and welfare status Any type of rehabilitation (sanatorium, polyclinic etc.), its duration and content (only asked at 6 months) Control of BP and cholesterol levels, experience of cardiac symptoms Frequency of physician consultations since discharge Diagnostic procedures since discharge Changes in smoking and alcohol habits Experience of counselling on risk factors Medications: names, doses, and frequencies. Changes of medications and reasons for doing so Availability of medications: receiving free, or paying own money, reasons for paying money Adherence to medicines
*Information extracted from medical records*
How the patient arrived at hospital and how long it took Date and time of onset of initial symptoms ECG results Troponin assay (whether conducted and results) Other investigations undertaken (including results), treatment provided following admission Revascularization procedures characteristics: type, date and time Blood pressure on admission Lipid profile and other laboratory tests Prior medical history Recommendations for follow up sent to polyclinic cardiologists at discharge *polyclinic cardiologists in Russia are general physicians with some specialist training in cardiology, they do not perform interventions
*Information extracted from polyclinic medical records*
**Twelve months prior to admission** Medical history and treatment as it relates to cardiovascular disease Consultations with primary care physician in 12 months prior to admission: number and content Blood pressure and cholesterol measurements Recommendations given in relation to cardiovascular risk factors, including lifestyle modifications and medications
**Twelve months following admission** Treatment recommended by general practitioner, comparison with recommendation from hospital and cardiologist, changes and reasons for changing (if recorded). Rehabilitation, BP and cholesterol measurement, Risk factors consultations etc.

Follow-up interviews at 6 and 12 months focus on self-reported treatment, medication, and contact with health services ( Box 3). As in all surveys, there is a trade-off between the length of the questionnaire, and thus the amount of data obtained, and the risk of respondent fatigue. Thus, while there are many instruments for assessing adherence to medicines, looking at patterns of adherence, reasons for non-adherence, and barriers to adherence ^18^, during piloting it became clear that an abbreviated set of questions was necessary. The single question “In the past month, how often did you take your medications as the doctor prescribed?” was used as it has been found to predict future cardiovascular events ^17^. This was supplemented with questions to identify reasons for non-adherence, including cost, belief that medicines are ineffective, and concerns about side effects, as well as forgetfulness. Questions on medications being taken were open, including the name of the medication, dosage and frequency. Responses were coded subsequently.

### Hospital medical records abstraction

Information on clinical management, both within the hospital during the baseline admission and in the ambulance in which the patient travelled to hospital, are obtained by abstraction of hospital clinical records ( Box 2) using a structured pro forma.

### Polyclinic medical record abstraction

As the study seeks to capture events across the entire patient journey, additional information is obtained from the polyclinic that the patient normally attends. In the Russian Federation individuals typically receive all continuing treatment at the same facility, either at where they live or work. The information required is extracted and recorded in two separate forms. The first relates to the 12 months prior to baseline admission. The second covers the 12-month period after that admission ( Box 2).

### Death verification procedures

Should a patient die it will usually be notified to the health facilities where they are receiving treatment. However, when participants are asked contact details they are also asked for details on a close relative, to help with tracing in case they are lost to follow up and not reported as dying. When deaths are identified, we will seek to obtain a copy of the medical death certificate from the health facility. For logistical reasons, it is not possible to undertake further adjudication of cause of death. However, over 50% of deaths in the Russian Federation are subject to autopsy ^19^, a much higher proportion than in many other countries.

### Progress with recruitment

As of February 2017, we had completed the baseline recruitment in all 13 regions ( Table 2). A total of 1,126 patients have been recruited. The number of subjects from each region varied from 12 to 128. The table also presents the number of patients who were hospitalized in 2015 with an AMI. The mean ages of subjects in each hospital are shown in Table 3. There is considerable variation in the proportion of patients who were dead on arrival or within 24 hours and among those who survived who agreed to participate across hospitals. 935 subjects have completed the 6-month questionnaire (83.0% response rate), 29 died within this 6-month period with 129 lost to follow up and 29 refused to participate.

**Table 2.** Recruitment of patients to the study.

	Estimated number of patients admitted with MI in six months †	Potentially eligible patients during recruitment period (based on eligibility scheme)	Died before first contact *		Not meeting inclusion criteria **		Refused to participate ***		Informed consent signed		
			N	% of potentially eligible patients recruited	N	% of potentially eligible patients recruited	N	% of potentially eligible patients recruited	N	% of potentially eligible patients recruited	% of all patients with MI admitted during recruitment
City hospital No 1, Archangelsk	294	89	0	0,0	12	13,5	11	12,4	66	74,2	22.4%
Altay regional cardiology hospital, Barnaul	181	93	1	1,1	0	0,0	31	33,3	61	65,6	33.7%
Belgorod regional hospital	250.5	105	2	1,9		0,0	7	6,7	96	91,4	38.3%
Bryansk cardiology clinic	127	77	4	5,2	0	0,0	2	2,6	71	92,2	55.9%
Kazan interregional clinic centre	215	154	5	3,2	7	4,5	15	9,7	127	82,5	59.1%
Kemerovo cardiology clinic	516	149	8	5,4	3	2,0	18	12,1	120	80,5	23.3%
Perm city clinic N 4	526	150	6	4,0	19	12,7	5	3,3	120	80,0	22.8%
Emergency city hospital, Rostov- on-Don	640	173	4	2,3	79	45,7	13	7,5	77	44,5	12.0%
Tver regional hospital	258	88	0	0,0	37	42,0	22	25,0	29	33,0	11.2%
Tver city hospital	159.5	38	6	15,8	2	5,3	2	5,3	28	73,7	17.6%
Saratov Regional cardiology hospital		22	3	13,6	0	0,0	7	31,8	12	54,5	
Samara regional cardiology clinic	1110	108	6	5,6	1	0,9	1	0,9	100	92,6	9.0%
Kinel rural hospital (Samara region)		21	0	0,0	0	0,0	0	0,0	21	100,0	
Otradny rural hospital (Samara region)		7	0	0,0	0	0,0	0	0,0	7	100,0	
Tuymen regional hospital	550	149	6	4,0	13	8,7	19	12,8	111	74,5	20.2%
Khanty-Mansiysk regional hospital	92	96	7	7,3	0	0,0	9	9,4	80	83,3	87.0%
Total	___	**1519**	**58**	3,8	**173**	11,4	**162**	10,7	**1126**	74,1	

* It is the patients who was considered eligible on the second day after hospitalization and for whom the task form were filled, but who died before first contact in the hospital (on the 2d or third and so on day of hospitalization, but not in 24 hours) ** It is mainly the patients for whom the initial diagnosis of MI was changed after 24 hours on other (angina and so on) and some special situation, when its was realized that eligible patient is a prisoner and it was decided after the consultation with central team to exclude him as follow up will not be possible. *** It is eligible patients which fit to inclusion criteria but which refused to participate in the study during the first contact and did not signed the informed consent † Estimated from annual total of admissions with myocardial infarction (all ages)

**Table 3.** Mean (standard deviation) age in years of study participants in each region.

Region	Males	Females	Total
Barnaul	57,6 ( 49,2 - 66,1)	65,6 ( 59,7 - 71,5)	60,1 ( 51,6 - 68,6)
Archangelsk	59,8 ( 52,1 - 67,5)	60,8 ( 52,6 - 69,0)	60 ( 52,2 - 67,7)
Belgorod	56,4 ( 47,4 - 65,3)	62,7 ( 54,6 - 70,8)	57,0 ( 48.0 - 66,1)
Bryansk	58,9 ( 51,1 - 66,6)	61,9 ( 55,8 - 67,9)	59,7 ( 52,3 - 67,1)
Kemerovo	56,8 ( 49,6 - 63,9)	61,4 ( 56,2 - 66,5)	57,9 ( 50,9 - 64,9)
Perm	58,3 ( 50,3 - 66,3)	62,4 ( 57,4 - 67,4)	59,2 ( 51,6 - 66,8)
Kazan	55,9 ( 47,4 - 64,5)	62,9 ( 55,8 - 70,0)	57,3 ( 48,6 - 66,0)
Rostov	59,0 ( 51,5 - 66,6)	60,9 ( 56,0 - 65,8)	59,5 ( 52,4 - 66,5)
Samara	57,9 ( 50,5 - 65,3)	61,2 ( 53,9 - 68,6)	58,8 ( 51,3 - 66,3)
Saratov	58,6 ( 52,1 - 65,1)	63,3 ( 59,8 - 66,8)	59,8 ( 53,6 - 65,9)
Tver	57,9 ( 51,3 - 64,5)	57,5 ( 52,1 - 62,9)	57,8 ( 51,4 - 64,3)
Tuymen	57,1 ( 48,7 - 65,5)	64,4 ( 59,1 - 69,8)	59,5 ( 51,3 - 67,8)
Khanty-Mansiysk	54,9 ( 45,6 - 64,1)	63,4 ( 56,3 - 70,4)	56,3 ( 46,9 - 65,7)
Total	57,4 ( 49,4 - 65,5)	62,4 ( 56,1 - 68,7)	58,5 ( 50,6 - 66,5)

### Analysis

This is, to our knowledge, the first attempt to understand the actual practice and management of AMI in the Russian Federation in both the acute phase in the critical year following the event using standardized methods. As such, the analysis will be exploratory, seeking to describe the extent of variation across a wide range of institutions in the case mix treated in each facility, including distances travelled to gain admission, and processes of care, such as investigations and treatments. We will also look at differences in diagnostic criteria or thresholds for diagnosing an AMI. Subsequent analysis will examine health seeking behavior, prior to the episode leading to admission, during that episode, and over the 12 months following the MI. This will include issues such as adherence to medicines and its determinants.

These exploratory analyses will generate a series of hypotheses that can be explored subsequently. For example, these could include testing hypotheses that patients are less likely to be treated with PTCA if they have certain characteristics. Thus, illustrative questions for analyses will include whether the patient’s gender or employment status influence their decisions in seeking care, found in research elsewhere, where men tend to disclose their symptoms as a means to receive help, whereas women tend to wait for others to discover their symptoms ^18^. Or do these characteristics influence clinical decisions, with patients who have certain characteristics treated differently? There are now many studies from other countries showing that, after taking account of differences in clinical features, older people ^19^ and women ^20, 21^ presenting with AMI are less likely to receive active treatment. Although all the facilities undertaking PTCA provide a 24-hour service, are there differences in management according to time of day, as has been found elsewhere, with differences in both case-mix and outcomes? ^22^

In testing such hypotheses, analysis will take account of the hierarchical structure of the data, with patients nested within facilities. Thus, where possible we will use multilevel models to take account of clustering of patients into hospitals and of hospital characteristics through the introduction of a random intercept (for the hospital) ^23^. These models will examine patient-level characteristics, such as distance from facility, gender and education, clinical characteristics, such as presenting symptoms or signs, and facility-level characteristics, introduced as fixed effects. Where appropriate, analysis will be stratified, for example by facilities offering PTCA or not.

## Discussion

 This study will, for the first time, provide detailed information on the AMI management in the Russian Federation, tracing the entire patient pathway and identifying variations in treatment, including both rates and delays, thereby elucidating barriers to effective management. Although it cannot claim to be nationally representative, it includes hospitals that span the entire spectrum of management in the Russian Federation, as judged by intervention rates. While recognising that the facilities we include may represent some of the better hospitals and clinics, in terms of staffing and facilities, our findings will still be of value. In this situation, they will provide an “upper” bound to the type and quality of care available to the bulk of the Russian population, at least outside of the large metropolitan centres of Moscow and St Petersburg.

There are some other sources of data on the management of AMI in Russia, including a federal registry, established in 2008 and including 213 clinics in 36 centres ^13^. We will draw on these data to supplement our interpretation of what we find. However, the federal registry includes only data on the index hospital stay and has several methodological problems, such as a lack of clear recruitment procedure and considerable missing data. Another source is a series of RECORD studies, collecting data on patients with acute coronary syndrome (ACS). The most recent, RECORD 3, was performed in the first 6 months of 2015 and included 2370 patients from 47 clinics. All patients with ACS hospitalized in the participating clinics in a single 1 month are recruited and although there is follow up over twelve months, these data are not available. Russian investigators also participate in the CLARIFY study ^24^, and have recruited 2,200 patients from across the country, but this collects different information than in the present study and included patients with stable forms of coronary heart disease.

Our study has strengths and weaknesses. First, although we can make no claim to be nationally representative, we do include a range of facilities in which AMI patients are treated and not only regional and academic centres. Moreover, we used a procedure to randomly select patients within facilities, this minimising the influence of subjective judgements that might have led to biases in the profile of cases. Second, we collect extensive data on pathways to care and follow-up not available elsewhere, including information that will enable us to gain insights into the reasons for any observed variations. However, we do not cover the entire country. In addition, our sample is based on willingness of cardiologists to participate. There is little tradition of clinician involvement in health services research in Russia, although we hope that this study will act as a catalyst to change this ^25^.

## Ethical statement

This main study and the pilot study was approved by the ethics committees at the National Research Institute for Preventive Medicine, Moscow, Russia (approval number 01-04/15 dated 03.02.2015) and at the London School of Hygiene & Tropical Medicine, London, UK (approval number 9993 dated 1 June 2015). All study participants signed informed consent to participate in the study, to grant the access to medical history and other medical documentation and to be contacted at 6 and 12 months after hospitalization.

## Data availability

No data is associated with this article.

## Supplementary material

Supplementary File 1: Rate of PTCA per 100,000 populations in all Russian regions in 2015 (official statistics, Ministry of Health, unadjusted crude rates).

.

Supplementary File 2: Baseline hospital survey questionnaire.

.

Supplementary File 3: Baseline hospital medical record extraction form.
