# Supplementary figures and images for: The management of acute myocardial infarction in the Russian Federation: protocol for a study of patient pathways
Source: Wellcome Open Res. 2018 Apr 6;2:89. Originally published 2017 Sep 25. [Version 2] doi: 10.12688/wellcomeopenres.12478.2 (PMC5930545; doi:10.12688/wellcomeopenres.12478.2)

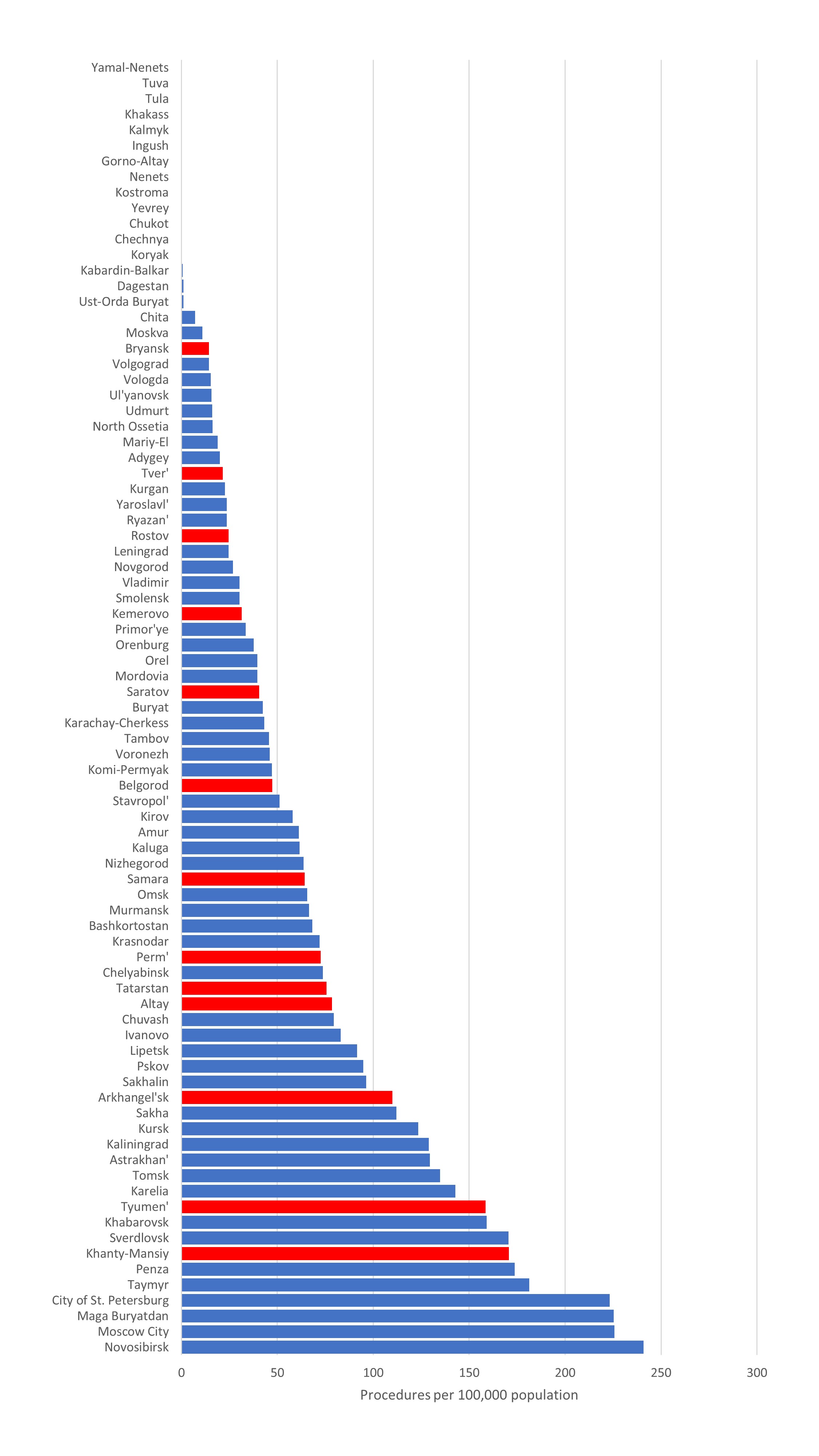

Supplement: Supplementary file 1 [file wellcomeopenres-2-15644-s0000.tgz › eba7667b-84c1-451c-b30e-b6d2a47d7738.jpg]
