# Supplementary material for: The management of acute myocardial infarction in the Russian Federation: protocol for a study of patient pathways
Source: Wellcome Open Res. 2018 Apr 6;2:89. Originally published 2017 Sep 25. [Version 2] doi: 10.12688/wellcomeopenres.12478.2 (PMC5930545; doi:10.12688/wellcomeopenres.12478.2)
Supplement: Supplementary file 2 [file wellcomeopenres-2-15644-s0001.tgz › 3e5aff91-b4f0-4b88-9c9b-6c38589d2332.pdf]

International Project on Cardiovascular Disease in Russia

Acute myocardial infarction in Russian Federation: current practice and barriers to effective treatment on different levels of healthcare

Baseline hospital survey

Baseline survey final, 10/06/15, NB

ACS study
Baseline hospital survey

	**Information about the interview**
**1.Date of the interview**	Day ______ Month______________Year
**2. Start time of the interview**	-------------------------------------
**3. Place of the interview**	Name of the medical facility<br><br>…………………………………………………………………….<br><br>1. In ward<br>2. In the office<br>3. Other<br>……………………………………………………………………….
**4. Who takes the interview**	
Last name	
First name	
Patronymic name	
**5.**	Are you...<br>*[Please select all that apply]*<br><br>1.Research fellow/researcher<br>2.Physician<br>3.Cardiologist<br>4 Other<br><br>Specify……………………………………….
**6. Interviewer ID**	**Region code_____Clinic number_____________Number**

	A. Participant information
**A1. Participant name**	
Last name	
First name	
Patronymic	
**A2.Address**	City/town………………………………………… Street…………………………………………………… House…………… Kor…………… Apt ……..
**A3. Telephone number**	Home…………………………………….. Mobile……………………………………. Tel number of a relative/friend_____________________ Name of the relative/Friend__________________________________ ____ ____ Tel number of a relative/friend_____________________ Name of the relative/Friend__________________________________ ____ ____
**A4. Date of birth**	Day ______ Month_______________Year
**A5. Sex**	1. Male 2. Female
**A6. The date of the index event (MI)**	Day ______ Month_______________Year

**A7. Place of hospitalization**	Name of the medical facility<br>……………………………………………………………………………………
**A8. Number/name of the policlinic where registered**	………………………………………………………………..<br><br>1.Not registered<br>2.Does not know<br>3. Other...<br><br>98
**A9. Participant ID**	**Region code_____Clinic number_____________Number**

Module B. Symptoms

B1. What symptoms were you having before you came to the hospital?

Choose all that apply
1 Chest pain, pressure, tightness or discomfort
2 Dizziness
3 Indigestion or stomach pain, pressure, burning or discomfort
4 Nausea
5 Pain or discomfort in jaw, neck, arm sore between your shoulder blades
6 Palpitations
7 Shortness of breath
8 Sweating
9 Weakness or fatigue
10 Confusion
11 Other symptoms
11a Specify other symptoms____
97 difficult to answer
98 refuse to answer

B2. Why did you decide to get help for these symptoms? ***[Check all that apply]***

1 Symptoms would not go away
2 Pain too bad to ignore
3 Worried about heart problem
4 Worried about other health problems (i.e. diabetes)
5 Family / friend told me to get help
6 Other
6a Specify other_______________________
97 difficult to answer
98 refuse to answer

B3. When you were first having these symptoms, did you think that something was wrong with your heart?

1 Yes ***Go to B5***
2 No
97 difficult to answer
98 refuse to answer

B4. What did you think was causing these symptoms?

1 Indigestion or acid reflux
2 Stomach illness

3 Flue
4 Muscle pain
5 Fatigue
6 Stress/anxiety
7 Asthma
8 Diabetes
9 Other
9a Other specify_________________________________
97 difficult to answer
98 refuse to answer

B5. What time these symptoms started?

Time………..Day………………………
1 Respondent reported exact time
2 Respondent reported approximate time
97 difficult to answer
98 refuse to answer

B5A Where symptoms appeared?

1 At home
2 At workplace
3 On the street
4 Other
4a Specify____
97 difficult to answer
98 refuse to answer

B5B Please specify the exact adress where you was when symptoms appeared
____

97 difficult to answer
98 refuse to answer

B6. When did you or someone for you call for help?

Time………..Day………………………

1 Respondent reported exact time

2 Respondent reported approximate time

97 difficult to answer

98 refuse to answer

B7. If you did not call for help immediately (withiin 15 minitues) what were the reason(s) that you decided to wait before seeking medical care? ***[Check all that apply]***

1 Didn't have time to go to the doctor

2 Symptoms did not seem bad enough for emergency care

3 Symptoms would come and go over time (not persistent)

4 Took medication for symptoms (i.e. antacids, over the counter meds)

5 Fear

6 Other ___________________________

6a Other speciy____

97 difficult to answer

98 refuse to answer

B8. When did you leave to the hospital?

Time………..Day………………………

1 Respondent reported exact time

2 Respondent reported approximate time

97 difficult to answer

98 refuse to answer

B8A Please specify the exact adress from which you was transferred to the clinic

____

97 difficult to answer

98 refuse to answer

B9. Did you visit a doctor for any of these symptoms before this hospitalization?

1 Yes

2 Never had these symptoms before **Go to B13**

3 Never visited your doctor for these symptoms ***Go to B13***

97 difficult to answer ***Go to B13***

98 refuse to answer ***Go to B13***

B10. How many times have you seen your doctor for these symptoms in the past 12 months?

Number of times……………………………………

97 difficult to answer

98 refuse to answer

B11. Did you see your doctor in the week before going to the hospital for any of these symptoms?

1 Yes

2 No

97 difficult to answer

98 refuse to answer

B12. Did your doctor ever tell you that your symptoms might be related to a heart problem?

1 Yes

2 No

97 difficult to answer

98 refuse to answer

B13. Where did you first go for help?

Choose all that apply

1 Called ambulance 03 or 112 on mobile phone
2 Went to the hospital by yourself or with help from relatives
3 Went to polyclinic
4 Went to the cardiologist
5 Other
5a Specify____________________________________
97 difficult to answer
98 refuse to answer

B14. Did you take aspirin before seeking medical attention (in this case)?

1 Yes
2 No, I did not have aspirin
3 No, I did not know I have to take aspirin
97 difficult to answer
98 refuse to answer

B15. Prior to your recent hospital stay, did you consider yourself at risk for heart disease or a heart problem?

1 Yes
2 No
97 difficult to answer
98 refuse to answer

B16. Prior to you recent hospital stay, did any of your healthcare providers ever tell you that you were at risk for heart disease or a heart problem?

1 Yes
2 No
97 difficult to answer
98 refuse to answer

Module C. Medical history. Now I will ask you about several health conditions you might have.

C1. **Have you ever been told by a doctor (been diagnosed) that you have:**

		Yes	No	97 DA	98 RA
1	Miocardial Infarction/Heart attack				
2	Heart failure				
3	Atrial fibrillation				
4	Peripheral artery disease				
5	Angina				
6	Stroke				
7	TIA (transient Ischaemic attack)				
8	Diabetes				
9	Kidney disease				
10	Chronic bronchitis/COPD				
11	Cancer				
12	Astma				

C2. **Have you ever been told by a doctor or other medical professional (been diagnosed) that you have high blood pressure?**

1 Yes
2 No ***Go to C4***
97 difficult to answer ***Go to C4***
98 refuse to answer ***Go to C4***

C3. **Have you taken medicine for high blood pressure in the past 12 months?**

1 Yes, always
2 Yes, sometimes
3 No

97 difficult to answer
98 refuse to answer

C4. **Have you ever been told by a doctor or other medical professional (been diagnosed) that you have high cholesterol?**

1 Yes
2 No ***Go to C6***
97 difficult to answer
98 refuse to answer ***Go to C6***

C5. **Have you taken medicine for high cholesterol in the past 12 months?**

1 Yes
2 No
97 difficult to answer
98 refuse to answer

(For those who reply “Yes” in C1, option 8)

C6. Have you taken medicine for diabetes in the past 12 months?

1 Yes
2 No
97 difficult to answer
98 refuse to answer

Module D. Use of health care services.

This module contains questions about use of medical services and use of medications

D1. **Where do you usually go for your health care?**

1 Did not need any health care
2 No particular place
3 District polyclinic
3a Specify number/name……………………………….
4 Policlinic at work
4a Specify number/name……………………………….
5 GP
6 Private clinic
7 Other
7a Specify ………………………………….

97 difficult to answer
98 refuse to answer

D2. **How many times you used the following sources of health care in the last 12 months? (Please check number of times for each type of doctor)**

Type of doctor	Number of times visited in last 12 months					
1.District physician	0	1	2	3	4	5+
2. Polyclinic cardiologist	0	1	2	3	4	5+
3. Other polyclinic specialist	0	1	2	3	4	5+
4. Hospital cardiologist	0	1	2	3	4	5+
5. Other hospital doctor	0	1	2	3	4	5+
6. Other……………………..	0	1	2	3	4	5+

D3. **In the last 12 months, how many times have you been hospitalised excluding this hospitalization (stayed in the hospital overnight)?**

Number of times..................................Number of days_________________

D4. **How many times have you called or someone called for you an ambulance in the last 12 months?**

Number of times.................

D5. **In the last 12 months, when you have visited a doctor (or any other medical professional), have you been advised to modify your lifestyle in any of the following ways to improve your health?**

**Change diet**	Yes	No	97/98
**Increase physical activity**	Yes	No	97/98
**Lose weight**	Yes	No	97/98
**Stop smoking**	Yes	No	97/98
**Reduce alcohol intake**	Yes	No	97/98

D5f **Did not visit doctors** **Yes**

D6. **Are you aware of the current polyclinic-based dispansarisation program in Russia?**

1. Yes
2. No ***Go to D10***

97 Difficult to answer ***Go to D10***

98 Refuse to answer ***Go to D10***

D7. **Have you received an invitation to participate in dispansarisation?**

1. Yes
2. No ***Go to D10***

97 Difficult to answer ***Go to D10***

98 Refuse to answer ***Go to D10***

D8. **Did you (or do you intend to) accept this invitation and attend dispansarisation?**

1. Yes ***Go to D10***
2. No

97 Difficult to answer ***Go to D10***

98 Refuse to answer ***Go to D10***

D9. **If not, why not?** *Check all that apply*

1. I don’t believe attending dispansarisation will have any positive effect on my health.
2. I feel well so I don’t need to get checked.
3. It is too far to travel to dispansarisation clinics.
4. The wait times at dispansarisation clinics are too long.
5. Dispansarisation clinics are not open at times that are convenient.
6. I do not know where I could attend dispansarisation

7 Other

7a Specify____

97 Difficult to answer

98 Refuse to answer

D10. Do you have any additional medical insurance with some other policlinic, medical centre, or/and insurance campany?

1. Yes
2. No
97 Difficult to answer
98 Refuse to answer

D11. What are the doses and frequency of used medications in the last 12 months prior to the index event? Please include any inhalers. Please start with the medications from the heart disease, high blood pressure or cholesterol and/or diabetes, which you are/were taking regularly.

Interviewer! Please, register up to 7 used medications. Dose of medication refers to strength of medication e.g. mg per tablet. If the respondent has difficulty to remember the name, dose or frequency, etc. please, write in option "97".

	Name of the medication	Dose (e.g. mg)	Number of units of medication used during one take	Frequency of use of medication
**D11d**			☐☐☐ tablets / capsules<br>☐☐☐ ml - injections<br>☐☐☐ drops	☐☐
**D11e**			☐☐☐ tablets / capsules<br>☐☐☐ ml - injections<br>☐☐☐ drops	☐☐
**D11f**			☐☐☐ tablets / capsules<br>☐☐☐ ml - injections<br>☐☐☐ drops	☐☐

Codes for frequency of use of medications:

01 daily 3 times a day
02 daily 2 times a day
03 daily once a day
04 3 times a week
05 2 times a week
06 once a week
07 fewer than once a week
97 don't know / difficult to

Module E. Smoking
I will now ask you some questions concerning your smoking habits

E1. Are you a current smoker?

Please circle the single most appropriate answer.

1 never a smoker ⇨**go to F1**
2 no, ex-smoker⇨**go to E2**
3 yes, a current-smoker **go to E3**

97 Difficult to answer
98 refuse to answer ***Go to F1***

E2. How many years ago did you stop smoking regularly?

Please circle the single most appropriate answer.

1 up to 1 year ago
2 more than 1, up to 5 years ago
3 more than 5, up to 10 years ago
4 more than 10 years ago
97 difficult to answer
98 refuse to answer ***Go to F1***

E3. How old were you when you started smoking regularly?

(open question)

		Years

97 difficult to answer
98 refuse to answer

E4. When you smoke/smoked, how many per day is/was usual?

Please circle the single most appropriate answer.

1 up to 10
2 more than 10, up to 20
3 more than 20
97 difficult to answer
98 refuse to answer

Module F. Alcohol

Now I am going to ask you some questions about your use of alcoholic beverages in the past year

F1. Have you consumed any alcoholic beverage in the past 12 months?

1 Yes
2 Do not drink at all ***Go to G1***

97 difficult to answer
98 refuse to answer

F2. Have you been drinking any alcohol drinks during the last 24 hours before this hopsitalization?
1 Yes
2 No

97 difficult to answer
98 refuse to answer

**F3. In the last 12 months, have you had any of the following experiences?**	**Yes**	**No**
Have you ever felt you should cut down on your drinking?	1	2
Have people ever annoyed you by criticising your drinking?	1	2
Have you ever felt bad or guilty about your drinking?	1	2
Have you ever had a drink first thing in the morning to steady your nerves or get rid of a hangover?	1	2

G. Socio-demographics. In this last part of the survey I will ask you several questions about yourself.

G1. What town, village or city do you live in?

……………………………………..

G2. How long have you lived in this place?

1 Less than 12 months
2 More than 12 months-up to 5 years
3 More than 5 years

97 difficult to answer
98 refuse to answer

G3. What is your current marital status?
1 Married
2 Divorced or separated
3 Widower
4 Single (never being married)

97 difficult to answer
98 refuse to answer

G4. Do you live… *Check all that apply*
1 Alone
2 with a spouse/partner
3 withchildren
4 with parents or parents-in-law
5 with siblings
6 other
6a Specify______________________
97 difficult to answer
98 refuse to answer

G5. What is your level of education?

Please choose the single most appropriate answer.

1 incomplete secondary
2 complete secondary
3 professional school (without secondary degree, PTU)
4 professional school and secondary (e.g. PTU and secondary education)
5 specialised secondary (e.g. technicum, college, medical, pedagogical college)
6 incomplete higher
7 Higher
97 difficult to answer
98 refuse to answer

G6. Are you….

1 Full-time employed
2 Part-time employed
3 Retired (exluding due to invalidity)
4 Retired because of invalidity
5 Unemployed
6 Other
6a Specify……………………………………………
97 difficult to answer
98 refuse to answer

G7 Do you have currently officialy registered disability?

1 Yes
2 No ***Go to G9***
97 difficult to answer
98 refuse to answer

G8. What is the class of the disability at the moment?

1 Class 1
2 Class 2
3 Class 3
97 difficult to answer
98 refuse to answer

G9. **Which of the phrases below best describes this household's financial situation during the past year?**

Please choose the single most appropriate answer.

1 There is not even enough money for food, it's difficult to make ends meet
2 We have enough money for food, but we find it difficult to afford clothes and other items
3 We have enough money for food and clothes, but would find it difficult to buy large domestic appliances
4 We can afford to buy large domestic appliances, but would find it difficult to buy a new car
5 We can afford to buy a large new car, but would find it difficult to buy a flat or a house (or other property)
6 We have no financial constraints. We can afford to buy a flat or a house (or other property).

97 difficult to answer
98 refuse to answer

G10. **How satisfied are you with your economic conditions at the present time?**

1 Fully satisfied
2 Rather satisfied
3 Both yes and no
4 Less than satisfied
5 Not at all satisfied

97 Difficult to answer
98 Refuse to answer

Thank you for answering my questions!

Section X: The final questions are about the circumstances of the interview

The following questions are answered only by you as an interviewer and are not to be read out:

X1 ***Time when the interview was completed***

…………………………………………………………..

X2. ***Were there any interruptions to the interview?***
1 yes
2 no ⇨***go to*** *X4*

X3. ***Please provide details of interruptions, including their duration:***

....
....
....

X4. ***Any other comments***

....
....
....
