# Supplementary material for: The management of acute myocardial infarction in the Russian Federation: protocol for a study of patient pathways
Source: Wellcome Open Res. 2018 Apr 6;2:89. Originally published 2017 Sep 25. [Version 2] doi: 10.12688/wellcomeopenres.12478.2 (PMC5930545; doi:10.12688/wellcomeopenres.12478.2)
Supplement: Supplementary file 3 [file wellcomeopenres-2-15644-s0002.tgz › 48ce084e-fe1f-49bc-92da-120e5f7b7a73.pdf]

International Project on Cardiovascular Disease in Russia

Theme C sub-study

Acute myocardial infarction in Russian Federation: current practice and barriers to effective treatment on different levels of healthcare

Baseline hospitalization medical record extraction form

Baseline hospitalization medical record extraction form

A. Study site information

**A1.Region**	
**A2.Hospital**	
**A3.Number of the hospital record**	
**A4.Date and time of data extraction**	**_______Day______Month______Year**<br><br>**Time____________________________**
**A5.Who extracted the data**	
Last name	
First name	
Patronymic name	
	Are you…<br>[Please choose all that apply]:<br><br>1.Research fellow/researcher<br>2.Physician<br>3.Cardiologist<br>Other___________
**A6. ID of the person who extracted the data**	**Region code___Clinic code____person number____**

B. Patient information

**B1. Patient full name**	
Last name	
First name	
Patronymic name	
**B2.Patient study ID**	**Region code___Clinic code____patient number____**
**B3. Date of birth**	___Day___Month___Year
**B4. Gender**	1 Male

	2 Female
**B5. Home address**	
**B6. Telephone number**	Home number Not indicated 99
**B6a**	Mobile number Not indicated 99
**B7. Next of kin name (relationship)**	Not indicated 99
Last name	
First name	
Patronymic name	
Relationship	
**B8. Next of kin tel number**	Not indicated 99

C. Hospital admission

**C1. Date**	___Day___Month___Year
**C2. Time**	___ : ___

**C3. Initial diagnosis**	1. Definite myocardial infarction<br>2. Acute coronary syndrome<br>3. Unstable angina<br>4. Other initial diagnosis<br><br>4a. Specify…………………………………………………

**C4. Symptom onset**	C4a Date ___Day___Month___Year<br><br>C4b Time ___ : ___

**C5. Call for help**	C5a Date ___Day___Month___Year<br><br>Not indicated 99

	C5b Time ___ : ___<br><br>Not indicated 99
**C6. Arrival of first responder**	C6a Date ___Day___Month___Year<br><br>Not indicated 99<br><br>C6b Time ___ : ___<br><br>Not indicated 99
**C7. Arrival of ambulance**	C7a Date ___Day___Month___Year<br><br>Not indicated 99<br><br>C7b Time ___ : ___<br><br>Not indicated 99
**C8. Admission method**	1. Direct admission via ambulance<br>2. Self-presenter to this hospital<br>3. Already in this hospital<br>4. Transfer from no-PCI center to PCI center<br>5. Repatriation after coronary intervention<br>6. From the policlinic (cardiologist referral)<br>7. Other<br>7a. Specify…………………………………………………..<br><br>Not indicated 99
**C9. Admission ward**	1. Not admitted as died in A&E<br>2. Cardiac care unit<br>3. General medical ward<br>4. Intensive therapy unit<br>5. Other<br>5a. Specify………………………………………………….<br><br>Not indicated 99
**C10. Admitting consultant**	1. Cardiologist<br>2. Resuscitator<br>3. Therapeutist

	4. Other general physician<br>4a. Specify…………………………………..<br>5. Other<br>5a. Specify…………………………………..<br><br>Not indicated 99
**C11. Symptom presentation**<br>*(Check all that apply)*	1. Typical Chest Pain (central chest pain, tight or crushing, and in some cases spreading into left arm or neck, best lying still)<br>2. Atypical Chest Pain (other type of chest pain or only radiation to arm and no effect of movement)<br>3. Back Pain<br>4. Abdominal Pain<br>5. Nausea and/or vomiting<br>6. Sweating<br>7. Shortness of Breath<br>8. Fatigue<br>9. Other symptoms<br>9a. Specify……………………………………………………<br><br>10. No symptoms<br><br>Place of first symptom presentation:<br>1. Home<br>2. Workplace<br>3. Street<br>4. Public place<br>5. Other, specify_______________
**C12. Where was aspirin given?**	1. Patient took it himself/herself<br>2. Aspirin/antiplatelet drug given in ambulance<br>3. Aspirin/antiplatelet drug given after arrival in hospital<br>4. Aspirin/antiplatelet drug contraindicated<br>5. Not given<br><br>Not indicated 99
**C13. Place first 12 lead ECG performed**	1. Home

	2. Ambulance car<br>3. In hospital<br>4. Other healthcare facility<br><br>Not indicated 99
**C14. Time first ECG**	C8a Date ___Day___Month___Year<br><br>C8b Time ___ : ___<br><br>Not indicated 99
**C15. ECG determining treatment**	***1.*** No acute changes ***Go to C18***<br>***[Or please select one or more of the suggestions below]***<br>2. ST segment elevation ***Go to C18***<br>3. Left bundle branch block ***Go to C18***<br>4. ST segment depression<br>5. T wave changes<br>6. Q wave<br>7. Other acute abnormality<br>7a. Specify…………………………………<br><br>Not indicated 99
**C16. QRS complex duration**	1. >=120 msec<br>2. <120 msec<br><br>Not indicated 99
**C17. Site of infarction based on ECG**	1. Anterior (V1-V6 ST elevation, No reciprocal ST dep)<br>2. Inferior (II, III, aVF elevation, reciprocal ST inversion in I, aVL)<br>3. Septal (V1-V4, no Q in V5-V6, no reciprocal ST dep)<br>4. Lateral (I, aVL, V5, V6 ST elevation, reciprocal ST dep in II,III, aVF)<br>5. Posterior (ST elevation: V7, V8, V9, reciprocal high R in V1-V3 with ST depression V1-V3 > 2mm)<br>***6.*** Indeterminate ***Go to C19***<br>7. No sign of definite MI in ECG ***Go to C19***

**C18.Patient location at when first ECG**	1. Onset of STEMI while patient not in hospital (STE on first ECG)<br>2. STE first recorded on subsequent ECG (non-interventional hospital)<br>3. STE first recorded on subsequent ECG (interventional hospital)<br>4. Not applicable<br><br>Not indicated 99
**C19.Systolic BP on admission**	…………………….mm Hg
**C20. Diastolic BP on admission**	………………………..
**C21. Heart rate**	……………………..Bpm<br><br>Type of rhythm:<br><br>1.Sinus rhythm<br>2.Atrial Fibrillation<br>3.Bundle branch block<br>4.Atrioventricular block (Grade 2 to 3)<br>5. Ventricular tachycardia
**C22. Killip class**	1. No evidence of heart failure (I)<br>2. Basal crepitations and/or elevated venous pressure (II)<br>3. Pulmonary oedema (III)<br>4. Cardiogenic shock (IV)<br>5. Not applicable<br><br>Not indicated 99
**C23. Weight**	…………………….Kg

**C24. Height**	…………………….Cm

D. Laboratory tests

(First = first available value within the first 24 hours)

**D1. Serum cholesterol (first)**	……………………..mmol/l
**D2. Lipids** (mmol/l; most recent in past 12 months)	Total serum cholesterol __ __ __ __<br><br>HDL __ __ __<br><br>LDL __ __ __<br><br>Triglycerides __ __ __ __ __<br><br>**D2a**. When obtained:<br>1. In hospital within first 24h<br>2. Prior to arrival (within past 12 months)<br>3. In hospital after first 24h<br><br>Not indicated 99<br><br>**D2b**.Fasting? 1 Yes 2 No Not indicated 99
**D3. Serum glucose (first)**	……………………..mmol/l<br><br>Fasting? 1 Yes 2 No Not indicated 99
**D4. HbA1c**	HbA1c__._ %<br><br>Not indicated 99
**D5. Creatinine (first)**	……………………..mmol/l

**D6. Haemoglobin (first)**	……………………..g/dl
**D7. Cardiac markers raised**	1.Yes<br>2.No<br>Not indicated 99
**D8. Troponin level (ng/mL)(circle type of Troponin for each)**	Time of **first Troponin**__________<br><br>• Troponin I • Troponin T First: __ __ __ __ . __ __ •
**D9. Troponin assay first**	1. Troponin I<br>2. Troponin T<br>3. High sensitivity Troponin T<br>4. High sensitivity Troponin I<br>99.Unknown
**D10. Troponin level (ng/mL)(circle type of Troponin for each)**	• Troponin I • **Troponin T Max**: __ __ __ __ . __ _
**D11.Troponin assay maximum**	1. Troponin I<br>2. Troponin T<br>3. High sensitivity Troponin T<br>4. High sensitivity Troponin I<br>99.Unknown
**D12. CK (IU/L)**	First: __ __ __ __ . __ Max: __ __ __ __ . __
**D12a. Corresponding CK-MB**	First: __ __ __ __ . __ Max: __ __ __ __ . __
**D13** eGFR ***[Please use CKD-EPI to calculated if not indicated]***	………………. ml/min/1.73m2

E. **Reperfusion** [opening artery with thrombolytic treatment, PCI or surgery]

**E1. Initial attempt of reperfusion treatment**	*Please mark only one option*<br>1. None Go to E6<br>2. Systematic Thrombolytic Treatment<br>3. Intracoronary Thrombolytic Treatment<br>4. PCI alone (primary POBA)<br>5. PCI with stent (primary PCI)<br>6. Referred for consideration for pPCI elsewhere<br>7. pPCI already was performed at the interventional hospital<br>8. Other<br>9a Specify………………………………….<br>99. Unknown
**E2. Thrombolytic drug**	1. Streptokinase<br>2. Alteplase<br>3. Tenecteplase<br><br>3a.Dose of Tenecteplase…………………………………………<br>4.Other<br>4a.Other TLT doses<br><br>5.None given<br><br>Not indicated 99
**E3. Where was initial reperfusion treatment given?**	1. No reperfusion attempted<br>2. Before admission to hospital in the ambulance<br>3. At the no-PCI center<br>4. In CCU (directly)<br>5. In other hospital ward<br>6. Cath lab<br><br>Not indicated 99
**E4.Reperfusion treatment date:**	Day___ / Months___ / Year___

**E4a. Reperfusion treatment time:**	___ : ___
**E5A: Was reperfusion first attempted within 3 hours of admission (or prior to admission)?**	1. Yes - go to E6<br>2. No
**E5B. Indicate all reasons why reperfusion was not performed within 3 hours of admission (or prior to admission)**	1.Sustained hypertension (systolic > than 180)<br>2.Clinical concern about recent cerebrovascular event or surgery<br>3.Delay obtaining consent<br>4.Initial ECG ineligible<br>5.Cardiac arrest<br>6. Ambulance 12 lead ECG not diagnostic of STEMI<br>7. Consideration of Primary PCI<br>8.Cath lab access denied<br>9. Delay in activating cath lab team<br>10. Pre-PCI complication<br>11.Equipment failure<br>12.Transfer from non-PCI center<br>13.Other<br>13a. Specify…………………………………………………………..
**E6. Was coronary angiogram performed?**	1. Yes<br>2. No ***Go to E13***<br><br>E6a Date ___Day___Month___Year<br><br>Not indicated 99<br><br>E6b Time ___ : ___<br><br>Not indicated 99
**E7.Was any coronary occlusion >50%**	1. Yes<br>2. No ***Go to E9***

	Not indicated 99
**E8. Select the vessels diseased (Tick all that apply):**	1. LM<br>2. LAD<br>3. LCX<br>4. RCA<br><br>5. Other<br>5a.Specify…………………………………………
**E9. Was any PCI or POBA attempted during the procedure?**	1. Yes<br>2. No ***Go to E13***<br><br>Not indicated 99
**E10. Did patient receive a stent?**	1. Yes<br>2. No ***Go to E13***<br><br>Not indicated 99
**E11. Drug eluting stent?**	1. Yes<br>2. No<br><br>Not indicated 99
**E12. Bare metal stent?**	1. Yes<br>2. No<br><br>Not indicated 99
**E13. Did patient get a CABG?**	1. Yes<br>2. No<br><br>Not indicated 99
**E14.Reason reperfusion treatment not given**	1. None<br>2. Ineligible ECG<br>3. Too late<br>4. Risk of haemorrhage<br>5. Uncontrolled hypertension<br>6. Administrative failure (ambulance or hospital)<br>7. Treatment postponed until later<br>8. Patient refused treatment<br>9. Late arrival to hospital (more than 24 hours)

	10.Other contraindications<br>10a. Specify………………………………………………..<br>11.Other<br>11a.Specify…………………………………………<br><br>Not indicated 99
**E15. Additional reperfusion treatment**	1. None<br>2. Repeated rescue PCI in the hospital<br>3. Referred for rescue PCI elsewhere<br>4. Facilitated PCI (PCI within 3 hours of initial TLT)<br>5. Additional dose of thrombolytic<br><br>Not indicated 99

F. Other procedures performed

**F1.Echocardiography**	1. Yes<br>2. No<br>3. Planned after discharge<br>Not indicated 99
**F2. Left ventricular ejection fraction?**	1. Good (>-50%)<br>2. Moderate (30-49%)<br>3. Poor (< 30%)<br>4. Not assessed<br><br>Not indicated 99

G. Previous medical history

**G1_1. Previous AMI**	1. Yes<br>2. No Go to G1_2<br>Not indicated 99
**G1_1a How long ago AMI?**	1. < 1 month<br>2. 1-6 months<br>3. > than 6 months
**G1_2. Previous Angina**	1. Yes<br>2. No<br>Not indicated 99
**G1_3. Hypertension**	1. Yes<br>2. No<br>Not indicated 99
**G1_4. Hypercholesterolaemia**	1. Yes<br>2. No<br>Not indicated 99
**G1_5. Peripheral vascular disease**	1. Yes<br>2. No<br>Not indicated 99
**G1_6. Stroke/TIA**	1. Yes<br>2. No<br>Not indicated 99
**G1_7. Asthma or COPD**	1. Yes<br>2. No<br>Not indicated 99
**G1_8. Chronic renal failure**	1. Yes<br>2. No<br>Not indicated 99
**G1_9. Previous PCI**	1. Yes

	2. No<br>Not indicated 99
**G1_10. Previous CABG**	1. Yes<br>2. No<br>Not indicated 99
**G1_11. Diabetes**	1. Yes<br>2. No ***Go t G2***<br>Not indicated 99
**G1_11a Diabetes control**	1. Dietary control<br>2. Oral Medicine<br>3. Insulin<br>4. Insulin plus oral medication<br>Not indicated 99
**G12. Smoking**	1. Never smoker<br>2. Ex-smoker<br>3. Current smoker<br>4. Smoking history unknown<br><br>Not indicated 99

H. Medications in hospital *(Please include all medications taken)*

	Name of the medication	Dose	Number of units of medication used during one take	Frequency of use of medication
**H1.**			tablets/ capsules<br>ml - injections<br>drops	
**H2.**			tablets/ capsules<br>ml - injections<br>drops	
**H3.**			tablets/ capsules<br>ml - injections<br>drops	

Codes for frequency of use of medications:

01 daily 3 times a day

02 daily 2 times a day

03 daily once a day

04 3 times a week

05 2 times a week

06 once a week

07 fewer than once a week

	Name of the medication	Dose	Number of units of medication used during one take	Frequency of use of medication
**H7.**			☐☐☐ tablets/ capsules<br>☐☐☐ ml - injections<br>☐☐☐ drops	☐☐
**H8.**			☐☐☐ tablets/ capsules<br>☐☐☐ ml - injections<br>☐☐☐ drops	☐☐
**H9.**			☐☐☐ tablets/ capsules<br>☐☐☐ ml - injections<br>☐☐☐ drops	☐☐

Codes for frequency of use of medications:

01 daily 3 times a day

02 daily 2 times a day

03 daily once a day

04 3 times a week

05 2 times a week

06 once a week

07 fewer than once a week

	I. **Hospital discharge**
**I1.Vital status**	1. Alive<br>2. Dead
**I2. Discharge/death date**	___Day___Month___Year
**I3. Total days in hospital**	Total days in hospital : __ __ __<br><br>If alive go to I12
**I4. (If dead) Is death…**	1. PCI Procedure-related<br>2. TLT related (ie bleeding before PCI)<br>3. Non-procedure-related<br>Not indicated 99
**I5. (If dead) Cause of death**	1. Cardiac<br>2. Vascular ***Go to I8***<br>3. Non-cardiovascular ***Go to I12***<br>Not indicated 99 ***Go to I12***
**I6. Is cardiac mode…**	1. Sudden<br>2. Non-sudden<br>Not indicated 99
**I7. Is cardiac death event caused by…**	1. AMI<br>2. Heart failure<br>3. Arrhythmia<br>4. Other<br>4a. Specify……………………….<br>Not indicated 99<br>***Go to I10***
**I8.Is vascular event…..**	1. Ischaemic stroke<br>2. Haemorrhagic stroke<br>3. Systemic haemorrhage<br>Not indicated 99
**I9. Is vascular event caused by…**	1. Peripheral embolism<br>2. Pulmonary embolism

	Not indicated 99
**I10.The cause of death certified by ……….**	1.Hospital pathologist<br>2.Forensic pathologist<br>3.Cardiologist<br>4.Other physician<br>………………………………………………………………
**I11.Cause of death as appear and coded on the death certificate**	…………………………………………………………
**I12. Physician's Final MI diagnosis**<br>*(select one)*	1. STEMI<br>2. NSTEMI<br>3. Acute MI of no clear type<br>4. Patient not diagnosed with MI ***Go to I14***
**I13. Location of MI**	1. Anterior (V1-V6 ST elevation, No reciprocal ST dep)<br>2. Inferior (II, III, aVF elevation, reciprocal ST inversion in I, aVL)<br>3. Septal (V1-V4, no Q in V5-V6, no reciprocal ST dep)<br>4. Lateral (I, aVL, V5, V6 ST elevation, reciprocal ST dep in II,III, aVF)<br>5. Posterior (ST elevation: V7, V8, V9, resiprocal high R in V1-V3 with ST depression V1-V3 > 2mm)<br>6. Other<br>6a.Specify…………………………………………………………..<br><br>Not indicated 99
**I14. Primary ICD-10 Discharge Diagnosis Code**	………………………………….
**I15. Secondary ICD-10 Discharge Diagnosis Codes**	………………………………………………………………………………<br>………………………………………………………………………………………<br>……………………………………………………………………………..<br>……………………………………………………………………………...<br>…………………………………………………………………………...
**I16.Patient instructions/education given**<br>*(choose all that apply)*	1. Discharge medications<br>2. Diet counselling<br>3. Activity guidelines<br>4. Out-patient cardiac rehab prescribed<br>5. Diabetes education<br>6. Lipid assessment planned

	7. Weight management counselling<br>8. Smoking cessation advice/counselling<br>9. Outpatient warfarin/anti-thrombotic therapy<br>10. Participated in in-patient cardiac rehab<br>11. Participated in rehabilitation in sanatorium<br>12. Other<br>12a.Specify………………………………………………………………..<br><br>99 Not indicated
**I15.Follow-up appointment scheduled with…**	1. Cardiologist<br>2. Cardiac surgeon<br>3. Terapevtist<br>4. Family practice physician<br>5. Endocrinologist<br>6. Policlinic physician<br>7. Other<br>7a. Specify………………………………………………………<br><br>8.Not indicated

J. Medications recommended after discharge

	Name of the medication	Dose	Number of units of medication used during one take		Frequency of use of medication
J1.				tablets/ capsules	
				ml - injections	
				drops	
J2.				tablets/ capsules	
				ml - injections	
				drops	
J3.				tablets/ capsules	
				ml - injections	
				drops	

Codes for frequency of use of medications:

01 daily 3 times a day

02 daily 2 times a day

03 daily once a day

04 3 times a week

05 2 times a week

06 once a week

07 fewer than once a week

	Name of the medication	Dose	Number of units of medication used during one take	Frequency of use of medication
J4			☐☐☐ tablets/ capsules<br>☐☐☐ ml - injections<br>☐☐☐ drops	☐☐
J5.			☐☐☐ tablets/ capsules<br>☐☐☐ ml - injections<br>☐☐☐ drops	☐☐
J6.			☐☐☐ tablets/ capsules<br>☐☐☐ ml - injections<br>☐☐☐ drops	☐☐

Codes for frequency of use of medications:

01 daily 3 times a day

02 daily 2 times a day

03 daily once a day

04 3 times a week

05 2 times a week

06 once a week

07 fewer than once a week

	Name of the medication	Dose	Number of units of medication used during one take	Frequency of use of medication
J7.			tablets/ capsules<br>ml - injections<br>drops	
J8.			tablets/ capsules<br>ml - injections<br>drops	
J9.			tablets/ capsules<br>ml - injections<br>drops	

Codes for frequency of use of medications:

01 daily 3 times a day

02 daily 2 times a day

03 daily once a day

04 3 times a week

05 2 times a week

06 once a week

07 fewer than once a week

Comments

………..
