# Supplementary material for: The management of acute myocardial infarction in the Russian Federation: protocol for a study of patient pathways
Source: Wellcome Open Res. 2018 Apr 6;2:89. Originally published 2017 Sep 25. [Version 2] doi: 10.12688/wellcomeopenres.12478.2 (PMC5930545; doi:10.12688/wellcomeopenres.12478.2)
Supplement: Peer review history

# Peer review: Referee response for version 2

**DOI:** 10.21956/wellcomeopenres.15644.r32880

**Published:** 2018-04-30

**Authors:** Robert Clarke

**Affiliations:** 1 CTSU (Clinical Trial Service Unit & Epidemiological Studies Unit), Nuffield Department of Population Health, University of Oxford, Oxford, UK

1. The tense is a mixture of present and past. It is best to describe all the protocol and data collected in the past tense.
2. Box 2 includes 6 month follow-up 2015-2917 (should be 2017)
3. Re-label Table 1 “selected characteristics of cardiology clinics in 13 regions of Russia in 2015”, add brackets round Total in second column header, and display all percentages to one decimal place (i.e. 82.0%).
4. Correct error in Figure 3. Policlinic (should be polyclinic).
5. Correct Table 2: (A) Remove decimal place for estimated number of patients (round up to whole numbers); (B) Replace commas by full stops for decimal points.
6. Correct Table 3: Replace commas by full stops, and display all values to one decimal place (i.e. 60.0).

Please find an edited PDF of the article here (edits highlighted in red).

I have read this submission. I believe that I have an appropriate level of expertise to confirm that it is of an acceptable scientific standard.

# Peer review: Referee response for version 2

**DOI:** 10.21956/wellcomeopenres.15644.r32879

**Published:** 2018-04-20

**Authors:** Ilmo Keskimäki, Sonja Lumme

**Affiliations:** 1 Department of Health and Social Care Systems, Social and Health Systems Research Unit, National Institute for Health and Welfare, Helsinki, Finland

We have now reviewed the revised manuscript and we feel that the authors have sufficiently revised the text and the tables and that all the comments we raised have been considered.

We have read this submission. We believe that we have an appropriate level of expertise to confirm that it is of an acceptable scientific standard.

# Peer review: Referee response for version 1

**DOI:** 10.21956/wellcomeopenres.13511.r28441

**Published:** 2017-12-21

**Authors:** Ilmo Keskimäki, Sonja Lumme

**Affiliations:** 1 Department of Health and Social Care Systems, Social and Health Systems Research Unit, National Institute for Health and Welfare, Helsinki, Finland

Review report on ”The management of acute myocardial infarction in the Russian Federation: protocol for a study of patient pathways” by Kontsevaya A, *et al*.

 1. Methods and study design / Objectives. We suggest that the overall objectives of the large study and objectives of this paper are clearly separated. Now the objectives describe mainly the aims of the large study and the aims of this paper remain indistinct.

 2. Supplementary File 1. How the rate is calculated? Is it a crude or age-adjusted rate? It is necessary to take into account the varying age differences between the regions. We do not fully understand these figures. Without any further explanation, the figure in the file seems to suggest that 55% of the population have undergone a PTCA in one region, which does not sound plausible. Or is the population somehow fixed, i.e. not the total population of the corresponding age group? The variation between the regions is also huge. We suggest explaining these differences at some extent. How accurate is the data from the official statistics?

 3. It is unclear, what is the proportion of potentially eligible AMI patients to all AMI patients in these facilities. It is important to know the (rough) estimate of the random sampling.

 4. Box 1. Inclusion and exclusion criteria: The exclusion criterion “Patients, referred from another facility…” is not self-explanatory for the readers who do not know the Russian health care system. Why exclude referred patients if the objective is to explore treatment pathways. Information on the (estimated) proportions of the patients included /exluded of the total AMI patient would be helpful, as well.

 5. Box 2. Study timeline: In the end date of the 6 months follow-up, there is an obvious mistake (Dec 2917).

 6. Figure 1 – an extra comma ”,” in the description of Selection.

 7. Page 5. What are the facilities for these four hospitals to perform PTCAs (which were not able to perform PTCAs 24h)?

 8. Table 1. Although this table describes the overall characteristics of these hospitals and not information on the study population, it would be informative to know the number of AMI patients in these hospitals. We recommend stating more clearly that this table does not describe the study population. It should be stated if the percentage of patients undergoing PTCA in this table refers to these AMI patients hospitalized in first 24 hours from symptom onset or if it is the overall proportion of performed PTCAs of all AMI or CHD patients.

 9. Figure 3. The second box in the lower row: policlinic or polyclinic

 10. Table 2. This table would benefit from some revision. It is somewhat confusing since there are numbers and percentages in disorder although it is mentioned in the column label. Perhaps percentage sign would clarify this.

 The definitions of the categories “not meeting inclusion criteria”, “refused to participate”, and “died before first contact” are unclear:

- If a potentially eligible patient has died before the first contact, why he/she is not categorized as a “not meeting inclusion criteria” patient (one of the inclusion criteria)?
- I understand these columns so that “not meeting inclusion criteria” and “refused to participate can be nested in some cases (Barnaul, Tuymen, Khanty-Mansiysk) or are the values incorrect for these hospitals? (Tuymen: 6+13+20+112=151, not 149)

 

 Values for Barnaul do not tally (93*0.667=62 NOT 55).

 11. Page 8, Progress with recruitment “The number from each region varied from 12 to…”. We suppose the correct number is 7.

 12. Analysis. Planned statistical methods to analyse this data are described only in the Abstract. We are concerned whether the number of this dataset will be enough for the multilevel modelling. There are only 7 cases in one region and 12 in the other and the number of covariate variables is large. Have you done some preliminary analyses to ensure that multilevel modelling is feasible?

We have read this submission. We believe that we have an appropriate level of expertise to confirm that it is of an acceptable scientific standard, however we have significant reservations, as outlined above.

# Response

**Published:** 2018-03-18

**Authors:** Anna Kontsevaya

**Affiliations:** therapy, Ivanovo state medical academy, Russian Federation

1. Methods and study design / Objectives. We suggest that the overall objectives of the large study and objectives of this paper are clearly separated. Now the objectives describe mainly the aims of the large study and the aims of this paper remain indistinct.

***Response: the objectives of the study have now been added to the text and a clear distinction between the larger study and paper objectives made (page 4 line 14)***

 2. Supplementary File 1. How the rate is calculated? Is it a crude or age-adjusted rate? It is necessary to take into account the varying age differences between the regions. We do not fully understand these figures. Without any further explanation, the figure in the file seems to suggest that 55% of the population have undergone a PTCA in one region, which does not sound plausible. Or is the population somehow fixed, i.e. not the total population of the corresponding age group? The variation between the regions is also huge. We suggest explaining these differences at some extent. How accurate is the data from the official statistics?

***Response: We thank the reviewer for pointing out an error in that the wrong graph was uploaded. This has now been corrected. The figures are the crude unadjusted rates (per 100 000 of population). These data are collected by regional health administrators and are believed to be accurate as they are the basis for payment and are carefully audited. However, it is not possible to calculate directly or indirectly standardized rates as they are not broken down by age.***

3. It is unclear, what is the proportion of potentially eligible AMI patients to all AMI patients in these facilities. It is important to know the (rough) estimate of the random sampling.

***Response: We did not systematically collect data on all patients admitted during the recruitment period. However, we do have information on the number of patients with MI admitted in 2015 and, from that, can estimate the number that would be admitted in a 6 month period (ignoring seasonal variation). We have now calculated the number recruited as a percentage of this figure. This ranges from 9% to 87% and is now shown in an additional column (last column on the right) in Table 2, for the 13 hospitals from which data from the Ministry of Health were available.***

4. Box 1. Inclusion and exclusion criteria: The exclusion criterion “Patients, referred from another facility…” is not self-explanatory for the readers who do not know the Russian health care system. Why exclude referred patients if the objective is to explore treatment pathways. Information on the (estimated) proportions of the patients included /exluded of the total AMI patient would be helpful, as well.

***Response We agree that a comprehensive assessment would include such patients. However, we faced several constraints. Most importantly, we did not have the capacity (in terms of trained clinicians willing to make the non-trivial commitment to participate and manage data collection in their hospitals) to include all hospitals in each region. This meant that we could not follow the entire pathway for all patients so we had to decide a point at which they entered the hospital system. We decided that this would most appropriately be their first admission to any hospital or a referral within 24hrs of the index event from another facility. By including a small number of rural hospitals, we then could begin to understand what types of patients are referred onwards to these larger centres, thus getting at least some idea about this group. Unfortunately, we do not have precise information on patients referred over 24 hours after the index event. However, within our sample, 21% of patients were transferred within 24 hours from a hospital offering no PCTA to a hospital within our study that does, 6% were referred from a cardiologist at a polyclinic within the first 24 hours.***

5. Box 2. Study timeline: In the end date of the 6 months follow-up, there is an obvious mistake (Dec 2917).

***Response: this has been corrected (page 6)***

6. Figure 1 – an extra comma ”,” in the description of Selection.

***Response: this has been corrected***

7. Page 5. What are the facilities for these four hospitals to perform PTCAs (which were not able to perform PTCAs 24h)?

***Response: They can provide thrombolysis. This has been clarified. The section now reads “Twelve (75%) of the facilities can perform PTCAs, all 24 hours a day; the remaining four are two small municipal hospitals in Samara, the regional cardiology hospital in Bryansk and one of the hospitals in Tver, all of which are able to perform thrombolysis).”***

8. Table 1. Although this table describes the overall characteristics of these hospitals and not information on the study population, it would be informative to know the number of AMI patients in these hospitals. We recommend stating more clearly that this table does not describe the study population. It should be stated if the percentage of patients undergoing PTCA in this table refers to these AMI patients hospitalized in first 24 hours from symptom onset or if it is the overall proportion of performed PTCAs of all AMI or CHD patients.

***Response: We have now added the number of AMI patients in 2015 and revised the text for clarity. We have also revised the table heading.***

9. Figure 3. The second box in the lower row: policlinic or polyclinic

***Response: We now use Polyclinic throughout for consistency***

10. Table 2. This table would benefit from some revision. It is somewhat confusing since there are numbers and percentages in disorder although it is mentioned in the column label. Perhaps percentage sign would clarify this.

The definitions of the categories “not meeting inclusion criteria”, “refused to participate”, and “died before first contact” are unclear:

- If a potentially eligible patient has died before the first contact, why he/she is not categorized as a “not meeting inclusion criteria” patient (one of the inclusion criteria)?
- I understand these columns so that “not meeting inclusion criteria” and “refused to participate can be nested in some cases (Barnaul, Tuymen, Khanty-Mansiysk) or are the values incorrect for these hospitals? (Tuymen: 6+13+20+112=151, not 149)

 Values for Barnaul do not tally (93*0.667=62 NOT 55).

***Response: The table has been reformatted and updated. Since the first submission some numbers have changed due to data cleaning. Now all the numbers sum to 100% and each patient can be only in one category.  We have clarified the definitions in a footnote***

11. Page 8, Progress with recruitment “The number from each region varied from 12 to…”. We suppose the correct number is 7.

***Response: We are referring to regions, not individual clinics here. Regionally, the lowest number was12 for the Saratov region7 it is the number from one clinic in Samara region, but there are 3 clinics there and total number for Samara region is 128. As a result, these numbers are correct and have not been changed here. *** 

12. Analysis. Planned statistical methods to analyse this data are described only in the Abstract. We are concerned whether the number of this dataset will be enough for the multilevel modelling. There are only 7 cases in one region and 12 in the other and the number of covariate variables is large. Have you done some preliminary analyses to ensure that multilevel modelling is feasible?

***Response; We thank the reviewers for raising this concern. We are planning, at the very least, multivariate analyses of the data. Multilevel modelling will form part of our analysis strategy, only where it is possible and the results obtained are of substantive interest and perform well in model diagnostic tests. Due to the nature of our data we absolutely do not expect to be able to incorporate any random slopes, for example, and thus this is not planned, we are most interested in a variance components (random intercept) model.***

***We are interested in the use of a random intercept both technically (in accounting for clustering and subsequent effects on precision of estimates) and substantively. We do not wish to run multilevel models unnecessarily and will consider how to stratify the sample for any multilevel analyses run to ensure they contribute to the overall aims of the study. Were multilevel modelling deemed inappropriate once model diagnostics are obtained we will look to the use of robust standard errors and/or fixed effects.***

***However, from a statistical perspective, using standard ‘rules of thumb’ we agree that any analyses with a regional intercept would more than likely require the exclusion of the Saratov region from the analysis (the lowest total number of patients is 12 (the region of Saratov) and we agree that this region would need to be excluded from any analyses that involved a random intercept, similarly the Samara hospital with 7 cases (and Saratov with 12) would also be excluded. We will also be considering ability to check normality assumption and non-zero variance when fitting these models, we strive to derive a technically appropriate substantively insightful analysis and there is a process of iteration in these planned analyses***

***We have adapted our references to the analysis strategy to highlight its multivariate nature, and the implementation of multilevel modelling where possible and in line with our substantive aims.***

# Peer review: Referee response for version 1

**DOI:** 10.21956/wellcomeopenres.13511.r26328

**Published:** 2017-10-16

**Authors:** Robert Clarke

**Affiliations:** 1 CTSU (Clinical Trial Service Unit & Epidemiological Studies Unit), Nuffield Department of Population Health, University of Oxford, Oxford, UK

The aim of this study is to publish a protocol for a study to evaluate the management of acute myocardial infarction (MI) in Russia. The study has recruited individuals aged 35-70 years from 16 hospitals across 13 regions in Russia. Individuals have to survive 24 hours after admission with a confirmed diagnosis of MI. The objectives of the study were to assess determinants of treatment decisions. Data were obtained on 1122 patients and the protocol acknowledges that the sample is not representative.

**Major comments:** 

1. Criteria for diagnosis of MI should reference WHO criteria. The current description of criteria is inadequate (e.g. no description of ST elevation, T-wave inversion, onset of Q-waves), elevated plasma levels of cardiac enzyme, etc. It would be best to specify a gold standard and see how many achieved this standard.
2. The impact of the requirement to survive 24 hours after presentation should be assessed.
3. It is unclear if only patients admitted to a coronary care unit or cardiology ward are eligible or whether the study involves all wards in any one hospital.
4. The study mentions specialized cardiology departments and general hospitals with cardiology departments, but does not specify if the difference is access to cardiac catheterization or cardiac surgery or other criteria. Selection bias may be relevant in the analysis.  
5. Table 1 provides characteristics of 13 hospitals but does not involve number of beds (measure of hospital size) or type of cardiology department (see Response 4.). Likewise, Table 1 should include mean age of study participants as the mortality may vary if the study clinic involves an older population.
6. Clarify what are polyclinic cardiologists (Box 3) and how these differ from cardiology departments.
7. The response rate was very low for Tver regional hospital, Rostov-on-Don and Saratov hospitals, but the reasons are unclear. It is also unclear why 78% of patients attending Rostov-on-Don were ineligible for the study. These discrepancies raise serious questions about the conclusions of this study.
8. The study report has multiple small grammatical errors that should be corrected prior to publication by careful review by a native English speaker. For example, the abstract includes a mixture of present and past tense when it would be best to limit it to past tense.
9. The data extracted from medical records should be used to confirm the diagnosis of MI.
10. The study did not mention whether there were written (online) protocols in place for management of AMI in some or all of these hospitals.

I have read this submission. I believe that I have an appropriate level of expertise to confirm that it is of an acceptable scientific standard, however I have significant reservations, as outlined above.

# Response

**Published:** 2018-03-18

**Authors:** Anna Kontsevaya

**Affiliations:** therapy, Ivanovo state medical academy, Russian Federation

1. Criteria for diagnosis of MI should reference WHO criteria. The current description of criteria is inadequate (e.g. no description of ST elevation, T-wave inversion, onset of Q-waves), elevated plasma levels of cardiac enzyme, etc. It would be best to specify a gold standard and see how many achieved this standard.
***Response: we obviously failed to clarify the aims of the study, and as requested by the other reviewer, have now done this. At the outset we felt that we could not be sure what diagnostic criteria were being used in Russian hospitals. Thus, one of our research questions was whether Russian clinicians were using WHO criteria. We now have the information that will allow us to answer that question. A secondary question was what happens to patients who have been given a diagnosis of AMI, regardless of whether it meets WHO criteria. This will then allow us to assess appropriateness of treatment. We hope we have now clarified this in the text.***
2. The impact of the requirement to survive 24 hours after presentation should be assessed.
***Response: The reviewer raises an important point but we believe that this would require a separate study. It is important to reiterate that this is the first study ever, to our knowledge, to study the management of AMI in Russian hospitals in a standardized manner, using established instruments. Even getting to this stage has been extremely challenging as there is very little (if any) tradition of health services research in Russia outside a few centres in the main cities. Thus, even when we have finished it there is a great deal that we will not know. The ideal way to answer this question would be to look specifically at those dying within 24 hours after admission, but this would require trained staff who could approach the families of patients in their own homes and collect sensitive information. We have some experience of doing this in our earlier research in Izhevsk, where we did collect data from family members of men who had died, but that project, in a single city, required a degree of effort that was an order of magnitude greater than what has been available to us here, including intensive supervision to ensure quality.***
3. It is unclear if only patients admitted to a coronary care unit or cardiology ward are eligible or whether the study involves all wards in any one hospital.
***Response: patients admitted to any ward of the clinic with primary diagnosis of MI were eligible, The presence of this diagnosis inevitably led to hospitalization either in a coronary care unit or an intensive care unit if one was unavailable. A sentence clarifying this has been added to the ‘Study population and recruitment’ section on pages 4-5.***
4. The study mentions specialized cardiology departments and general hospitals with cardiology departments, but does not specify if the difference is access to cardiac catheterization or cardiac surgery or other criteria. Selection bias may be relevant in the analysis.  
***Response: The facilities have been selected to include a range of those that exist in Russia. One of the questions we will be asking is to what extent the availability of facilities influences practice, although we concede that the study has limited power to go far in this direction. We are not sure if the reviewer means selection bias in terms of who goes where. If so, we suspect this will not be an issue as in all but a few very large cities there is no real choice. We have added a sentence on cardiac surgery in the ‘selection and characteristics of facilities’ section on page 7***
5. Table 1 provides characteristics of 13 hospitals but does not involve number of beds (measure of hospital size) or type of cardiology department (see Response 4.). Likewise, Table 1 should include mean age of study participants as the mortality may vary if the study clinic involves an older population.
***Response: We have added columns with numbers of number of beds (total and cardiology) to Table 1. We have also added a separate, new tablewith mean (and SD) age ( Table 3).***
6. Clarify what are polyclinic cardiologists (Box 3) and how these differ from cardiology departments.
***Response: We realise that we should have clarified this and have added some words. In brief, in Russia, there are physicians who have received training in a speciality, but at a very basic level. They are employed in polyclinics but their skills are not commensurate with what one would expect in the West and most would have the training in a specialist area possessed by a GP elsewhere (but they would not work outside that speciality). For example, they would undertake basic assessments of ECGs etc. and provide medical management of angina. In contrast, those working in cardiology departments in hospital would have a high level of training, including in areas such as interventional cardiology (which is separate subspecialty which requires separate training). We have now clarified this in box 3 (page 11)***
7. The response rate was very low for Tver regional hospital, Rostov-on-Don and Saratov hospitals, but the reasons are unclear. It is also unclear why 78% of patients attending Rostov-on-Don were ineligible for the study. These discrepancies raise serious questions about the conclusions of this study.
***Response: We agree that this is a potential problem. We have been unable to elicit reasons for these differences and, once the data have been fully cleaned, we will examine them carefully for any unusual features, as well as considering the use of sensitivity analyses when we come to analyse the data.***
8. The study report has multiple small grammatical errors that should be corrected prior to publication by careful review by a native English speaker. For example, the abstract includes a mixture of present and past tense when it would be best to limit it to past tense.
**Response: *We have been through the paper again carefully and we hope that any issues have now been resolved. ***
9. The data extracted from medical records should be used to confirm the diagnosis of MI.
**Response: This is being done. We are sorry if we did not clarify this in the text, which we now do. Figure 3 includes the point about hospital record extraction and box 3 details of the data extracted.**
** **
10. The study did not mention whether there were written (online) protocols in place for management of AMI in some or all of these hospitals.
**Response: We agree that we should have mentioned this (in fact, we have another paper under review which describes in detail the process by which guidelines have been developed in Russia). The relevant document covering the period when these patients were treated is the Federal clinical guideline approved by Ministry of health in 2013.** **http://mzdrav.rk.gov.ru/file/mzdrav_18042014_Klinicheskie_rekomendacii_Ostryj_infarkt_miokarda.pdf**
**This has now been added.**
